# Molecular Pathways and Druggable Targets in Head and Neck Squamous Cell Carcinoma

**DOI:** 10.3390/cancers13143453

**Published:** 2021-07-09

**Authors:** Farzaneh Kordbacheh, Camile S. Farah

**Affiliations:** 1 Department of Medical Oncology, Dana-Farber Cancer Institute, Boston, MA 02215, USA; Farzaneh_Kordbacheh@DFCI.HARVARD.EDU; 2 ACRF Department of Cancer Biology and Therapeutics, The John Curtin School of Medical Research, Australian National University, Canberra, ACT 0200, Australia; 3 The Australian Centre for Oral Oncology Research & Education, Perth, WA 6009, Australia; 4 Genomics for Life, Brisbane, QLD 4064, Australia; 5 Anatomical Pathology, Australian Clinical Labs, Subiaco, WA 6008, Australia; 6 Peter MacCallum Cancer Centre, Head and Neck Cancer Signalling Laboratory, Melbourne, VIC 3000, Australia

**Keywords:** head and neck cancer, squamous cell carcinoma, molecular pathways, biomarkers, personalised medicine, druggable targets, molecular therapies

## Abstract

**Simple Summary:**

Head and neck cancer remains a significant burden on patients and global health systems. Traditional approaches to therapy have included surgery, and more recently radiation and chemotherapies. Targeted immunotherapies are making significant inroads into improved outcomes, but only for small subsets of patients. Our ability to develop a wider range of targeted therapies rests on our understanding of the molecular pathways involved in carcinogenesis. This review paper summaries our current knowledge of the molecular pathways and druggable targets in head and neck oncology.

**Abstract:**

Head and neck cancers are a heterogeneous group of neoplasms, affecting an ever increasing global population. Despite advances in diagnostic technology and surgical approaches to manage these conditions, survival rates have only marginally improved and this has occurred mainly in developed countries. Some improvements in survival, however, have been a result of new management and treatment approaches made possible because of our ever-increasing understanding of the molecular pathways triggered in head and neck oncogenesis, and the growing understanding of the abundant heterogeneity of this group of cancers. Some important pathways are common to other solid tumours, but their impact on reducing the burden of head and neck disease has been less than impressive. Other less known and little-explored pathways may hold the key to the development of potential druggable targets. The extensive work carried out over the last decade, mostly utilising next generation sequencing has opened up the development of many novel approaches to head and neck cancer treatment. This paper explores our current understanding of the molecular pathways of this group of tumours and outlines associated druggable targets which are deployed as therapeutic approaches in head and neck oncology with the ultimate aim of improving patient outcomes and controlling the personal and economic burden of head and neck cancer.

## 1. Introduction

Head and neck cancers (HNC) and their treatment can result in significant morbidity for patients. Patients are often diagnosed at advanced stages with lymph node metastasis. Therefore, the diagnosis of HNC at early cancerous stages has become vitally important. A greater majority of HNC are squamous cell carcinomas (HNSCC) arising from the epithelial tissue of the oral cavity, pharynx and larynx [1]. Numerous genetic aberrations are involved in head and neck tumours. Additionally, various environmental factors including HPV infection and tobacco and alcohol exposure are associated with HNSCCs. HNSCC is a highly heterogeneous tumour type, hence personalised patient management should be based on both patient and tumour features [1].

HNSCC has traditionally been considered an environmental tumour mostly caused by tobacco and alcohol consumption and human papillomavirus (HPV) infection in Western populations [2,3,4]. There is evidence to suggest a critical role for genetic alterations contributing to the carcinogenesis of HNSCC [5]. This is demonstrated by a growing proportion of younger low-risk patients with suggested poorer prognosis and a distinctive clinical and histopathological pattern [6,7], in addition to genetic and prognostic differences between HPV-positive and HPV-negative HNSCC [8].

Currently the optimal treatment for HNSCC patients centres around a multidisciplinary approach including medical and surgical subspecialties such as surgery, medical oncology, radiation oncology, in addition to allied health disciplines. Targeted treatments have benefitted only a small subgroup of patients due to intrinsic and extrinsic drug resistance with limited drug efficacy [9]. This effect decreases with long-term follow-up [10]. Therefore, identification of new molecular targets and novel pharmacotherapies, as well as better patient selection, calls for new methods of identification and clinical validation of biomarkers.

In the past decade, massively parallel sequencing, also known as next generation sequencing (NGS) has enabled unbiased cancer genome sequencing in order to screen and search for new cancer genes at an unprecedented scale. NGS has been widely implemented for de novo whole genome, exome, and transcriptome sequencing for assessment of DNA copy number, re-arrangements, loss of heterozygosity, allele-specific amplification, methylation, transcription, aberrant splicing and RNA editing faster and more cost effectively than Sanger-based sequencing [11,12,13]. In recent years, NGS has bridged the gap between molecular screening and clinical applications with 96.1% accuracy in comparison to Sanger sequencing. In addition, it can reveal gene alterations at very low allelic frequency [14]. Not only has NGS helped identify new altered genes for novel biomarker development [15,16]; but by revealing specific gene alterations it has helped identify patients who are sensitive or resistant to particular therapies [17]. Genomic alterations collected by the International Cancer Genome Consortium (ICGC) and The Cancer Genome Atlas (TCGA) projects, in addition to the Catalog of Somatic Mutations in Cancer (COSMIC) database provide the most comprehensive source of somatic mutations in cancer to date.

Most known cancer genes have been found through primary cytogenetic analyses, although sequencing of cancer genomes has revealed new cancer genes including *BRAF*, *EGFR*, *ERBB2*, *PIK3CA*, *PPP2R1A*, and *JAK2* [18,19,20,21,22,23]. Specifically in relation to HNSCC, Agrawal and Stransky independently and simultaneously confirmed previously known HNSCC genome alterations such as mutations in *TP53*, *CDKN2A*, *PIK3CA*, *PTEN*, and *HRAS* [24,25] but also identified a novel gene *NOTCH1* [15,16]. *NOTCH1*, an important tumour suppressor gene, is second most common gene involved in HNSCC [26], and had not been identified by Sanger sequencing due to its large size (34 coding exons), despite it being previously shown to be important in functional studies in cutaneous SCC [27].

More targeted approaches have also been undertaken to explore the cancer genome of HNSCC. Mahjabeen et al. sequenced 17 exons of *XRCC1* in HNC patients and matched controls, and found two silent mutations in 45% and two missense mutations in 55% of cases. This accounted for a total mutation frequency of 87%. These silent mutations were distributed equally among smokers and non-smokers and amongst males and females [28]. Scheckenbach and colleagues sequenced exons and adjacent introns of *RAD51C* and showed five distinct heterozygous sequence alterations in 5.8% of HNSCC cases [29]. Transcriptional profiling of oropharyngeal SCC and matching normal samples showed *TP53* mutation along with CHEK2 and ATR over-expression (both p53 DNA damage repair pathway gene targets) in HPV-negative current smokers compared to past or non-smokers [30].

Given the heterogeneous nature of HNC generally and HNSCC specifically, there is great expectation that targeted therapies underpinned by deeper understanding of the molecular oncological pathways in these tumours, will result in better outcomes for patients. The promise of more effective therapeutic options for patients suffering from these tumours cannot be overstated, given the poor outcomes associated with advanced HNSCC. The potential of molecular profiling is tremendous, with the underlying goal of achieving “genome-informed personalised medicine” where a family-based disease history, gene expression pathways, and drug resistance and toxicity is considered in formulating a treatment plan [31]. Understanding of the mutational and genomic landscape of HNSCC [32] has enabled better use of therapies such as EGFR inhibitors and, more recently, immune checkpoint inhibitors [33], and will continue to drive innovations in targeted druggable approaches. This paper summarises the molecular pathways and druggable targets involved in head and neck cancer as a precursor to understanding current and emerging therapies in head and neck oncology, as detailed by us elsewhere [34].

## 2. Molecular Pathways and Druggable Targets

### 2.1. EGFR Pathway

Epidermal growth factor receptor (EGFR/HER1/ErbB1) is a 170-kDa transmembrane glycoprotein and one of four members of ErbB receptor tyrosine kinase family. Other members include HER2 (ErbB2), HER3 (ErbB3) and HER4 (ErbB4). EGFR has an important role in initiating the signalling that directs the behaviour of epithelial cells and tumours of epithelial origin. The EGFR signalling pathway is crucial in mammalian cells and regulates proliferation, migration, differentiation, and apoptosis as well as intercellular signalling during development. Binding of ligands and growth factors including EGF, transforming growth factor (TGF)-α and amphiregulin induces both hetero- (i.e., EGFR-HER2) and homo-dimerisation (EGFR-EGFR) of tyrosine kinases, which leads to their intracellular phosphorylation and activation of downstream signal transduction cascades [35,36]. Signalling proteins which have src homology-2 (SH2) or phosphotyrosine binding (PTB) domains can bind to the EGFR family members after tyrosine phosphorylation. The most important activated pathways are Ras/Raf/MEK/mitogen-activated protein kinase (MAPK)/ERK, phosphatidylinositol 3-kinase (PI3K)/AKT/mammalian target of rapamycin (mTOR), and Janus kinase (JAK)/signal transducer and activator of transcription 3 (STAT3) pathways [37,38]. These pathways are implicated in tumour cell growth/survival, proliferation, local invasion, angiogenesis, metastasis, protein translation and cell metabolism [38,39]. EGF-bound EGFR can also translocate to the nucleus to function as a transcription factor and one of its nuclear targets, *CCND1*, encodes cyclin D1 which is involved in cell cycle progression [40]. Aberration of EGFR signal activation, transduction, duration and intensity can result in disruption of cancer cell homeostasis.

EGFR family members are involved in a wide range of human diseases from psoriasis, cancer, inflammation, heart diseases to Alzheimer’s disease. Mutations in EGFR have been associated with adenocarcinoma of the lung, glioblastoma, and head and neck epithelial tumours. Mutations leading to overexpression lead to constant activation and consequently uncontrolled cell division, and its family members are implicated in about 30% of all epithelial cancers. Over expression of EGFR and its hetero-dimerisation with HER2 have been shown to be of prognostic value in HNSCC [41,42,43,44]. EGFR overexpression is mainly at the transcriptional level, as there is only up to 7% and 30% *EGFR* gene point mutation and amplification reported in HNSCC cases, respectively [44,45,46,47]. A mutant variant of EGFR (EGFRvIII) with 2–7 exons missing in the extracellular region has been reported in 42% of HNSCC patients [48]. Interestingly, *EGFR* gene copy number has not been found to be an efficient biomarker for EGFR-directed therapy [49] and it has been associated with poorer prognosis and patient outcome [49,50]. Hence EGFR and HER2 are feasible targets for cancer therapy, and agents targeting these molecules have been FDA-approved for HNSCC treatment, demonstrating increased response rates and increased overall survival when combined with standard therapy [51].

### 2.2. PI3K/AKT/mTOR Pathway

The PI3K/AKT/mTOR signalling pathway is one of EGFR downstream signalling networks and plays an essential role in cellular regulatory mechanisms including cell growth, differentiation, survival, proliferation, migration and glucose metabolism [52,53,54]. The PI3K family of lipid and protein kinases is divided into three classes, Class I, II and III, based on their primary structure, regulation, and lipid substrate specificity; but only Class I kinases function as second messengers in intracellular signalling pathways and are most frequently associated with oncogenesis. Class I PI3K is a heterodimer protein comprised of two main subunits (with several isoforms) of 110-kDa (p110) and 85-kDa (p85) which mediate enzyme catalytic and regulatory activity, respectively [35,55]. The p110 subunit exists in three isoforms, p110 α (encoded by *PIK3CA*), β (encoded by *PIK3CB*) and δ (encoded by *PIK3CD*), while the p85 subunit is encoded by three genes, *PIK3R1* (p85α), *PIK3R2* (p85β) and *PIK3R3* (p85γ) [55].

Following activation by receptor-associated tyrosine kinases (RTKs) such as EGFR, the catalytic subunit phosphorylates 3′-hydroxyl group of phosphatydilinositol 1,4-bisphosphate (PIP_2_) to form phosphatydilinositol 1,4,5-triphosphate (PIP_3_). Then, PIP3 calls pleckstrin-homology domain-containing proteins including phosphoinositide-dependent protein kinase 1 (PDK1) and AKT to the plasma membrane which results in phosphorylation of AKT by PDK1 and mammalian target of rapamycin complex 2 (mTORC2). Activation of AKT and, consequently, mTORC1 activates ribosomal protein S6 kinase 1 (S6) and inactivates eukaryotic translation inhibition factor 4E-binding protein 1 (4E-BP1) resulting in protein synthesis, cell growth and proliferation [35,56]. However, the tumour suppressor phosphatase and tensin homology (PTEN) tightly regulates the cellular level of PIP_3_ by converting it to PIP_2_ through its lipid phosphatase activity, and therefore counteracting the activation of AKT and its downstream pathways [57].

Genetic aberrations of the PI3K/AKT/mTOR pathway are very common in head and neck cancers [58]. In particular, as genome sequencing findings in 2011 revealed, pathway activation is frequently (6% to 20% of HNSCCs) mediated by mutations in *PI3KCA* which codes for p110α with more than 80% of mutations occurring in exon 9 (helical domain) as well as mutations in exon 20 (kinase domain) and exon 4, especially through the mechanisms of gene amplification and low-level copy number increase [15,16]. In addition, copy number increase in 3q26 has been reported as a common and early oncogenic event in almost half of HNSCC cases [59,60] which has been associated with a more invasive phenotype [61], vascular invasion [62] and higher chance of lymph node metastasis [60]. Additionally, *PTEN* mutations have been reported in 10% of HNSCC cases [63]; however, mutations may not be the primary mechanism for *PTEN* loss in HNSCC [64]. Moreover, the mTOR pathway can be independently activated from EGFR activation or mutant p53 presence, especially in patients with HPV-positive tumours [58].

### 2.3. RAS/RAF/MEK/MAPK Pathway

The RAS/MEK/ERK (MAPK) signal transduction pathway activates many important cellular mediators leading to cell growth, proliferation, differentiation, migration, invasion and survival [65,66,67]. Ras is a guanosine nucleotide binding protein (GTPase) localised at the intracellular side of the plasma membrane and is activated by TRKs including EGFR. The *Ras* gene family consists of three members, *HRAS* (Harvey), *KRAS* (Kristen) and *NRAS* (neuroblastoma) [26,35,68]. Once Ras becomes activated, it converts from guanosine diphosphate-bound (GDP-bound) inactive state to guanosine triphosphate-bound (GTP-bound) active state which activates RAF (RAF1) pathway as well as PI3K pathway [69]. RAF exists in three isoforms including ARAF, BRAF and CRAF (RAF1) [70]. Although they all activate MEK, it has been shown that they might be differentially activated by oncogenic RAS [71]. Activated RAF phosphorylates MEK1 and MEK2 kinases that, in turn, activate MAP kinases ERK1 and ERK2, which may either phosphorylate cytoplasmic targets or translocate into the nucleus to target genes affecting mechanisms such as cell growth, proliferation and survival [26,35,72].

Oncogenic RAS mutations are implicated in almost 30% of all cancer types (http://cancer.sanger.ac.uk, accessed on 7 February 2021) [69] which prevents switching of GTP and GDP, keeping RAS in its constitutive GTP-bound active state and consequently active downstream signalling results in proliferation and survival [73]. *KRAS* mutations have been reported for almost 90% of pancreatic adenocarcinomas, occurring mainly in exon 12 as well as in colorectal and lung cancers while *HRAS* and *NRAS* mutations are rarely seen in those conditions. However, *NRAS* and *HRAS* mutations are frequently seen in melanoma and salivary gland tumours, respectively [74]. The work of Stransky and Agrawal independently revealed that *HRAS* mutations are one of the most common mutations (6th and 8th) and were found in 4–6% of HNSCC cases [15,16].

Among all RAF mutations, *BRAF* mutations have been associated with many cancers including nervous system, melanoma and thyroid (http://cancer.sanger.ac.uk, accessed on 7 February 2021). One of the most common *BRAF* mutations is a single-base missense substitution of T to A at nucleotide 1799 in exon 15 (known as hot spot), that substitutes valine for glutamic acid at amino acid 600 (V600E) in the kinase domain of protein which results [75,76] in impaired kinase activity in a RAF1-dependant manner and generating kinase resistance to feedback inhibition and activating the MAPK pathway [77]. In 2003, Weber et al. performed direct DNA sequencing on 89 HNSCCs and showed that somatic *BRAF* mutations were relatively rare (approximately 3%; 3/89 cases), and all mutations were in exons 11 and 15. In addition to BRAF V600E mutations, *BRAF* mutations in 1403 nucleotide, resulting in substitution of G to T and therefore replacing alanine with glycine, were detected. They also found that heterozygous mutations of *KRAS2* occur in 6% of HNSCC within exons 12 and 13. Their results confirmed that *KRAS2* and *BRAF* mutations do not co-exist in HNSCC; hence, oncogenic *KRAS2* activates wild-type *BRAF*, but mutated *BRAF* does not require *KRAS2* for activation, suggesting simultaneous mutations may be redundant [65].

### 2.4. NOTCH Pathway

As with PI3K/AKT and MAPK, NOTCH pathway is a conserved signal transduction cascade which affects cell function such as self-renewal capacity, cell differentiation and survival in a cell- and context-specific manner [35,78]. The NOTCH family consists of four receptors which are bound to the cell membrane (NOTCH1-4) and interact with two families of ligands including Delta-like (Dll1, DllL3, Dll4) and Jagged (Jag1 and Jag2) ligands [79]. Ligand binding to NOTCH receptors leads to NOTCH cleavage by TNFα-converting enzyme (TACE) and γ-secretase which releases the NOTCH intracellular domain (NICD). The NICD consists of several domains including JM, RAM, anykrin repeats (ANK) and transcriptional activation domain (TAD); however, TAD is lacking for Notch 3 and Notch 4. Adjacent to the carboxyl-terminus of the NICD lies the PEST domain, which can be ubiquitinated by the FBXW7-containing E3 ubiquitin ligase complex resulting in NICD destruction [78]. The NICD regulates transcription of target genes such as *HRT* and *HES* families through the NOTCH ANK and NOTCH RAM motif interactions with transcriptional machinery partners, including the CBF1, Su(H), Lag-1 (CSL) DNA-binding transcription factor and Mastermind transcriptional coactivators (MAML1–3), respectively [80,81,82,83].

So far, a dual biological activity of Notch signalling has been reported in solid tumours. Preliminary reports of *NOTCH1* mutations in T cell acute lymphoblastic leukaemia (TALL) and chronic hematopoietic cancers were implicated as oncogenic [84,85,86,87]. Genome sequencing of HNSCC suggested *NOTCH1* acts as a tumour suppressor gene, and was the second most frequently mutated with an incidence of 15–19% [15,16]. In 2016, several *NOTCH1* somatic mutations were identified using whole-exome sequencing and were validated in a 13-year cohort of 128 HNSCC patients [88].

Recent integrated analysis has identified the deficiency of NOTCH pathway in 66% of HNSCC patients [89]. NOTCH1 signalling promotes terminal differentiation of keratinocytes and is negatively regulated by EGFR pathway. EGFR-activated c-jun suppresses p53 and NOTCH1 whereas blockade of EGFR induces keratinocyte differentiation in cutaneous SCC [90]. Moreover, in basal epithelial cells, NOTCH1 has been inhibited by p53-related transcription factor p63 (TP63) where its downregulation during terminal differentiation results in NOTCH1 upregulation. Overexpression and amplification of TP63 has been reported in many HNSCC cases [91]. Additionally, it has been observed that *NOTCH1* is significantly reduced in HPV-positive cervical carcinoma cells as expression of activated NOTCH1 results in strong growth inhibition of these cells via down-modulation of transcription of the E6/E7 viral genes. Thus, NOTCH1 expression is likely to play a protective role in late stages of HPV-induced carcinogenesis [92]; however, its role in HPV-induced HNSCC should be investigated further.

Not only can *NOTCH1*, *NOTCH2* and *NOTCH3* (3–5% of cases) mutations contribute to modulate the NOTCH pathway [15,16], but JAG1, JAG2, MUMB and MAL1 chromosomal aberrations have been reported to be involved as well [89]. *FBXW7* mutations have been identified in 5% of HNSCC for the first time, mainly in a hotspot known to block the degradation of active *NOTCH1* [15,16]. It is noteworthy that mutations in *FBXW7* may also target other cancer-related proteins including cyclin E and c-myc [15].

### 2.5. MET Pathway

Mesenchymal-epithelial transition factor (MET) is a receptor tyrosine kinase (RTK) which is encoded by *c-MET* proto-oncogene located on chromosome 7q21-q31 [93,94]. MET contains several functional domains including juxtamembrane (JM) (regulatory domain), receptor tyrosine kinase domain (TK) and semaphorin (SEMA) which binds to its ligand, hepatocyte growth factor (HGF) [95,96]. MET-HGF binding induces MET dimerisation, autophosphorylation and activation of TK catalytic activity which activates other cellular signalling pathways including RAS/RAF/ERK, PI3K/AKT/mTOR, JAK/STAT and NOTCH which result in cell growth, motility and survival [94,96,97,98].

Overexpression of MET has been detected in solid tumours such as 57% of SCC [99,100] which has been associated with enhanced cell motility, angiogenesis and invasion/metastasis as well as aggressive phenotype and poor prognosis [95,96]. MET/HGF overexpression and increased MET copy number have been found in over 80% and 13% of HNSCC cases, respectively [101,102,103]. HPV-positivity of head and neck tumours is usually accompanied by p16 positivity and can have very different outcomes when compared to HPV-negative cancers [104]. Although in some studies Met expression has been reported to be a prognostic biomarker in HPV-negative HNSCC [105,106], others have found no statistically significant correlation between c-Met positivity and p16 positivity [103].

MET mutations for HNC have been reported mostly in the TK domain with up to 25% of lymph node metastasis association [96,107]. Although a large number of mutations in JK domain have been detected for cancers including small-cell lung cancer (SCLC) [99,108], not many reports are available for mutations in SEMA and JM domains in HNSCC [102].

### 2.6. JAK/STAT Pathway

The Janus kinase/signal transducer and activator of transcription (JAK/STAT) signalling pathway mediates mechanisms including cell growth, proliferation, differentiation, survival, angiogenesis and inflammatory/immune responses via transmitting signals from the plasma membrane to the nucleus [109]. The JAK family is a non-receptor tyrosine kinase and consists of four members including JAK1, JAK2, JAK3 and tyrosine kinase 2 (TYK2) which each has seven regions known as Janus homology domains 1 to 7 (JH1-7). The JH1 domain is located at the C-terminal end of the protein and has tyrosine kinase activity containing conserved tyrosines (e.g., Y1038/Y1039 in JAK1, Y1007/Y1008 in JAK2, Y980/Y981 in JAK3, and Y1054/Y1055 in TYK2). The JH2 has tyrosine kinase structure but does not have the enzymatic activity (pseudokinase domain) and it regulates JH1 activity. The JH3–JH4 domains share homology with Src-homology-2 (SH2) domains. The JH4-JH7 domains are assumed kinase domains with JH6 and JH7 domains (N-terminal of JAK) being associated with cytokine receptor binding [110,111,112].

The STAT proteins are a family of transcription factors with seven identified members, STAT1, STAT2, STAT3, STAT4, STAT5A, STAT5B and STAT6 which each has six conserved domains including oligomerisation domain at the N-terminal of protein, coiled coil domain, DNA binding domain, linker domain, SH2 domain and transactivation domain at the C-terminal. Additionally, all STATs have an essential tyrosine near SH2 domain which is critical for their dimerisation, nuclear translocation and DNA binding. Moreover, STAT1, 3 and 5 can also be phosphorylated at a C-terminal serine (Y727 in STAT3) to achieve their maximum transcriptional activity [113]. Extracellular binding of cytokines to their receptors induces activation of the intracellular JAK activation to recruit and phosphorylate STAT proteins resulting in STAT dimerisation via their SH2 domain and then translocation to the nucleus to activate transcription of their target genes. JAKs can also be phosphorylated directly by RTKs such as EGFR and also G-protein-coupled receptors (GCPRs) to activate downstream signal cascades including RAS/MAPK and PI3K/AKT pathways [114,115]. STATs can also be activated independently of JAKs by growth factor receptors such as activation of STAT1, 3 and 5 by EGFR [116,117,118] and STAT5 by the platelet-derived growth factor receptor (PDGFR) [119]. Additionally, STAT3 can be activated by non-receptor tyrosine kinases such as c-Src and Abl [120,121].

Among all STATs, STAT1, 3 and 5 have been most implicated in tumorigenesis including in head and neck cancers [122]. Activation of STAT3 stimulates tumour-associated angiogenesis via modulating the stability and activity of hypoxia-inducible factor-1α (HIF-1α) as well as enhancing VEGF expression by binding to the VEGF promoter with HIF-1α [123,124]. STAT3 also promotes cell invasion/metastasis by activating the transcription of matrix metalloproteinase 1 (MMP1), MMP2, MMP9, and MMP10 [117,125,126,127,128]. In addition, STAT3 can suppress p53 expression [129] and expression of pro-inflammatory cytokines and chemokines necessary for immune-mediated tumour rejection including IL-12, TNF, IFN-γ, and IFN-β [130,131].

The SOCS1-7 protein family, also known as STAT-induced STAT inhibitors (SSIs), are members of cytokine-inducible negative regulators of cytokine signalling affecting JAK/STAT pathway by JAK activation and constitutive signalling [132]. In lung cancer, SOCS-3 acts as a growth suppressor and apoptosis inducer [133]. Methylation of SOCS-1 has been shown in 65% of hepatocellular carcinoma (HCC) cases (Yoshikawa et al., 2001). Hyper-methylation of SOCS-1 and SOCS-3 promoters was shown to be correlated with SOCS-1 and SOCS-3 gene silencing in HNSCC samples [134,135]. Increased EGFR signalling and STAT3 activation and overexpression has been detected in HNSCC [136]. STAT3 has also been found to be a Src-dependent mediator of EGFR-stimulated growth of HNSCC in vitro and decreased apoptosis and increased tumour growth in vivo [136,137]. Src family kinases can contribute to gastrin-releasing (GRP)-mediated EGFR growth and invasion pathways by facilitating cleavage and release of TGF-α and amphiregulin in HNSCC [138,139]. However, another study showed that after durable Src inhibition in HNSCC cell lines, STAT3 was reactivated through a compensatory pathway via altered JAK-STAT3 binding and JAK kinase activity [140].

Furthermore, activation of STAT5 has been shown to enhance tumour growth, invasion and epithelial-mesenchymal transition in HNSCC [141]. It was demonstrated that HNSCC cell invasion requires STAT-5A, but not STAT-5B. The activation of STAT-5A can be activated independent from JAK2, but with EGFR activity [142]. Conversely, HNSCC proliferation in vitro and in vivo requires STAT-5B but not STAT-5A [143,144]. Additionally, erythropoietin has been found to mediate activation of STAT5 through JAK2 and contribute to cellular invasion in HNSCC [145]. Moreover, it was shown that after Src inhibition which diminishes SOCS2 expression, STAT5A-mediated SOCS2 expression regulates JAK2 /STAT3 activity and survival signals [146].

### 2.7. TP53/RB and HPV-Mediated Pathogenesis

The proliferation of mammalian cells is a strictly regulated event and genetic aberrations in each part of this multistep process may confer tumorigenic effects. P53 transcription factor and retinoblastoma protein (RB) are two prominent tumour suppressors which are also assumed as cell cycle regulators [147]. The TP53 gene (*TP53*) is located on the short arm of chromosome 17 (17p13.1) and is involved in many events including glucose metabolism in cancer cells, and DNA-repair and apoptosis [148,149]. The level of p53 is regulated by mouse double minute 2 homolog (MDM2) which binds to the N-terminal of protein and after its ubiquitination degrades p53 as E3 ubiquitin protein ligase [150]. Conversely, *CDKN2A* encodes for p14 (also known as ARF and INK4B) which inhibits MDM2 and protects p53 from degradation [147,151]. Moreover, ataxia telangiectasia mutated (ATM) and ataxia telangiectasia and Rad3-related (ATR) pathways detect DNA damage and activate p53 via phosphorylation of the cell cycle checkpoint kinases CHK1 and CHK2 [152,153]. Activation of p53 results in induction of other cell cycle regulatory and apoptotic genes and proteins including *BAX* and *PUMA* [151,154,155] and p21 (cyclin-dependent kinase inhibitor 1 (CDKN1)) [156,157], p63 and p73 [158,159].

The RB protein is encoded by the *RB1* gene which is located at 13q14.1-q14.2 and prevents the cell division with damaged DNA through G1 restriction by preventing its progression from G1 (first gap phase) to S (synthesis phase) [160]. RB binds to and inhibits E2F transcription factor family from processing through the cell cycle. The mitogenic signals activate cyclin and cyclin-dependent kinases (CDK) including cyclin D1/CDK4/CDK6 complex which results in inactivation of RB via phosphorylation to pRB, followed by additional phosphorylation by cyclin E/CDK2 complex. Cyclin D1-CDK4/6 complex is normally inhibited by p21 (CDKN1) and p16 (also known as INK4A, MTS1 and CDKN2) which is encoded by *CDKN2A*. RB phosphorylation results in release of E2F and cell cycle transition to S, G2 and M phases [147,161].

*TP53* mutations are the most common genetic alterations in HNSCC (50–80%), mostly in exons 5 to 8 which encode the DNA binding domain of the protein (L1-L2 region) [15,162,163]. Mutations can also be detected as an early event in premalignant dysplastic lesions and histopathologically negative tumour surgical margins [164,165,166]. p53 has been associated with increased risk of progression from mild dysplasia to invasive carcinoma [165,167,168]. These mutations can confer either loss of tumour suppressor function or gain of function as a dominant oncogene to promote tumorigenesis via different pathways [169,170]. Genome sequencing data of HNSCC cases have revealed that 50–63% of *TP53* mutations are missense with the remainder (16% nonsense, 16% insertion/deletion, and 8% splice site mutations) predicted to be inactivating mutations [15,16]. According to TCGA, 69.8% of HNSCC showed *TP53* mutations, which makes HNSCC the third most mutated p53 carrier after ovarian cancer and lung squamous cell carcinoma [171]. In 2020, a computational analysis of TP53 mutational landscape using TCGA database showed that 286 HNSCC patients exhibited 129 different kinds of *TP53* mutations [172].

p53, RB, p16 and p14 inactivation through mutation, deletion or epigenetic silencing as well as cyclin D1 (*CCND1* gene), MDM2 and CDK4 overexpression have been associated with tumorigenesis and/or reduced survival in HNSCC [147,151,162,173,174]. Loss of heterozygosity (LOH) of p16 located at 9p21 has been reported in 30% and up to 80% of premalignant and malignant lesions, respectively [175]. While LOH in chromosome 3p has also been shown to be involved in HNSCC [176], co-occurrence of LOH in 3p and 9p can promote differentiation of dysplastic and hyperplastic lesions that are likely to progress to carcinoma [175,177,178]. Moreover, sequencing data have shown that inactivation of *CDKN2A* is associated with mutations and copy number loss found in 7–9% and 20–30% of HNSCC cases, respectively [15,16]. However, it has been shown that *CDKN2A* inactivation via mutation is less common than deletions or epigenetic inactivation, which together cause up to 75% of gene inactivation in HNSCC [179,180].

Activation of wild type p53 and RB in HNSCC can be inhibited by other mechanisms including human papillomavirus (HPV) infection. HPV is a small non-enveloped DNA virus which has been associated with carcinogenesis in both male and female sexual organs [181]. HPV infection has also been identified as a major cause of oropharyngeal squamous cell carcinoma (OPSCC) [182]. The HPV genome consists of eight open reading frames which encode two late (L1 and L2) capsids and six early (E1–E7) proteins [183,184]. Among more than 100 identified HPV genotypes, types 16, 18, 31, 33 and 45 (high risk HPV) have been most frequently associated with epithelial cell malignant transformation, with type 16 implicated in over 90% of HNSCC cases [181,185]. HPV E6 and E7 oncoproteins are able to engage with p53 and RB protein selectively. HPV E6 interacts with E6-associated protein (E6-AP) and promotes p53 ubiquitin proteasome degradation [186,187]. HPV E7 protein competes with E2F for binding to RB, and since RB acts as a negative regulator for the cyclin-dependent kinase inhibitor p16, overexpression of p16 has been found to be of great clinical value for determination of HPV-positive status using immunohistochemistry (IHC) [188,189]. It is noteworthy that HPV-negative samples may also reveal p16 expression through *RB1* mutation [190,191] which makes p16 staining a less reliable marker [192]. In addition to HPV E6/7 contribution to tumorigenesis in HPV-positive patients, recent sequencing data have shown that HPV-positive and negative oropharyngeal carcinomas cluster into two different subsets of genetic alterations [15,16,190]. HPV-negative HNSCC show *TP53* mutations in almost all samples as well as *CCND1* amplification and *CDKN2A/B* deletion in approximately 50% of tested cases. HPV-positive HNSCC however, show more frequent *PIK3CA*, *PTEN*, *FBXW7* and *KRAS* gene alterations but almost no *TP53* mutations [32,190].

### 2.8. Cell Cycle Pathway

The eukaryotic cell cycle leads to cell growth and division and consists of two major steps, interphase and M phase. Interphase is a series of events in the cell cytoplasm and nucleus in preparation for cell division and consists of G1, S and G2 phases. Interphase lasts for 91% of the cell cycle. M phase includes mitosis and cytokinesis. Cell chromosomal separation occurs in mitosis, which is immediately followed by cytokinesis, which divides the nuclei, cytoplasm, organelles and cell membrane into two cells with an equal share of cellular components. M phase accounts for approximately 10% of the cell cycle. Two important classes of regulatory molecules, cyclins and cyclin-dependent kinases (CDKs) are key regulators of the cell cycle.

The G1 phase includes D- and E-type cyclin elevation, activation of cyclin-dependent kinases (CDKs), phosphorylation of the retinoblastoma protein (pRb), and activation of the E2F transcription factor family [32]. In G2 phase, additional cyclin/CDK complexes are activated including cyclin B1/CDC2 complex which stimulates mitotic entry [193].

Cyclin D1 (CD1) is a member of cyclin family, which includes CD1, CD2 and CD3 (also known as CDK4). CD1 is a 45-kDa protein encoded by the *CCND1* gene on chromosome 11q13. Amplification of *CCND1* and overexpression of cyclin D1 have been reported in almost 50% and 80% of OSCCs, respectively [194]. Overexpression of cyclin D1 leads to shortening of the G1 phase and increases independence of growth factors and abnormal cell proliferation, which fosters the occurrence of additional genetic alterations [33]. *CCND1* amplification and cyclin D1 overexpression in HNSCCs have been reported to be associated with a high rate of relapse, lymph node metastasis and shorter patient survival [194].

Cell division protein kinase 6 (CDK6) is an enzyme encoded by *CDK6* gene and is regulated by cyclins, more specifically by Cyclin D proteins and Cyclin-dependent kinase inhibitor proteins. This kinase is a catalytic subunit of the protein kinase complex, important for G1 phase progression and G1/S cell cycle transition. CDK6 and CDK4, phosphorylate and thus regulate the activity of tumour suppressor Retinoblastoma protein, making CDK6 an important protein in cancer development [1].

Aberrant expression of CDK6 protein has been observed in oesophageal and oral SCC [195]. Overexpression of CDK6 was observed in OSCC cell lines as well as OSCC tissue compared with normal oral mucosa and oral leukoplakia tissues with or without dysplasia [195]. CDK6 overexpression in HNSCC was significantly correlated with T classification and resulted in tumour progression [31].

The tumour suppressor *CDKN2A* was identified as the second most commonly altered gene in HNSCC in TCGA [63]. *CDKN2A* (also known as P16, INK4, p16INK4A, and MTS1) is allelic to chromosome 9p21 and encodes a CDK4/CDK6 kinase inhibitor that constrains cells from progressing through the G1 restriction point. It is thought to be involved in the early stages of HNSCC development. It is affected in up to 80% of HNSCC; often deleted, hyper-methylated, or, more rarely, mutated [196]. *CDKN2A* mutation is considered “noncoding mutation”, “inactivation”, or “loss of function” [197]. It is associated with worse overall survival in patients with recurrent and metastatic HNSCC [63]. Most mutations in *CDKN2A* are found in exon 2 [196]; however, these are likely insufficient to drive tumorigenesis in their own right [26]. Therapeutic targeting of *CDKN2A* presents a challenge of restoring tumour suppressor activity or inhibiting downstream targets that have been rendered overactive [198].

### 2.9. DNA Repair Pathway

DNA repair is a series of events by which a cell identifies and corrects DNA damage. Cell cycle checkpoints are activated following DNA damage. Checkpoint activation pauses the cell cycle and gives the cell time to repair the damage before continuing with division. DNA damage checkpoints occur at the G1/S and G2/M boundaries. Checkpoint activation is controlled by two master kinases, ATM (ataxia–telangiectasia mutated) and ATR (ataxia–telangiectasia and Rad3 related) alongside BRCA1, BRCA2, MDC1, and 53BP1 [199]. These proteins are required for transmitting the checkpoint activation signal to a downstream signal transduction cascade and result in cell cycle arrest [200]. Additionally, PALB2 (partner and localiser of BRCA2) interacts with BRCA1 and BRCA2 and plays pivotal roles in DNA repair [201].

Although *BRCA1* and *BRCA2* are among the top 30 of 236 most commonly altered genes in HPV-negative HNSCC [202], somatic mutations in other genes involved in DNA damage in HNSCC are seen at varying frequencies [202]. The frequency of somatic mutations in *BRCA1* (6%), *BRCA2* (7%), *ATR* (4–10%) and *ATM* (1–16%) support the rationale for targeting components of the DNA repair pathway as druggable targets in HNSCC [203].

### 2.10. Hypoxia and Angiogenesis

In solid tumours, oxygen supply to cells is reduced or abolished due to microvessel structural abnormalities, disturbed microcirculation, and high oxygen consumption associated with high metabolic activities of tumour cells, resulting in tumour hypoxia [204,205]. Tumour hypoxia is a common event in HNSCC and is associated with poor prognosis, treatment resistance and reduced survival in patients [206,207]. Hypoxia-inducible factors (HIFs), including HIF-1α and HIF-2α are ubiquinated and degraded under normoxic conditions by Von Hippel– Lindau protein (VHL). Hypoxia results in HIF stabilisation and heterodimerisation with HIF-2β and translocation into the nucleus act as transcription factors [35,208]. HIF-1α targets genes including phosphoglycerate kinase (PGK), glucose transfer 1 (GLUT1), carbonic anhydrase 9 (CA9), and vascular endothelial growth factor (VEGF) [209,210], while HIF2α mediates EGFR activation [211]. HIFs are partially regulated by mTOR pathway as they show sensitivity to mTOR inhibitors [212].

VEGF (VEGF-A) plays a critical role in angiogenesis and is a member of the platelet-derived growth factor (PDGF) superfamily along with VEGF-B, VEGF-C, VEGF-D, VEGF-E and placental growth factor (PIGF) members [213]. VEGF ligands act through their cell surface tyrosine kinase receptors, VGFR1 (Flt-1), VGFR2 (KDR/Flk-1) and VGFR3; with VGFR2 being the most important receptor involved in proliferation, differentiation and migration of vascular endothelial cells along with VEGF [214]. As with hypoxia, overexpression of VEGF in HNSCC has been associated with more advanced disease, increased resistance to cytotoxic agents and poor prognosis as well as more aggressive phenotype [215].

In addition to VEGF overexpression caused by HIF, hypoxia affects various cellular pathways in different ways which may result in opposing outcomes. For example, hypoxic conditions enhance VEGF mRNA stabilisation by binding proteins to its 3′ untranslated region (UTR) [216] as well as post-transcriptional regulation of VGFR2 while inducing interaction of VEGF-soluble VGFR1 by overexpression of VGFR1 which inhibits VEGF action [217,218]. Moreover, hypoxia can induce apoptosis in oncogenically transformed cells with HPV E6/E7 or c-myc genes [219,220]. Furthermore, it has been shown that p53 levels increase in hypoxic conditions which induce apoptosis influencing Apaf1 and caspase9 pathways [221]. Hypoxia can also promote apoptosis via a p53-independent pathway, involving HIF-1 and downregulation of expression of the anti-apoptotic BCL-2 family [222,223].

Dysplastic oral tissue displays higher expression of angiogenic cytokines such as VEGF, IL8/CXCL8, FGF2, and HGF compared to normal samples. In HNSCC, there may be two potentially distinct pathways involved in angiogenesis. Samples showed either higher levels of VEGF and FGF2 which were associated with higher angiogenic index, or expressed lower levels of VEGF and FGF2 with higher levels of IL8/CXCL8 and HGF [224]. The multiplicity of HIF induced angiogenesis mechanisms implies that not only is the same angiogenesis phenotype a result of different molecular mechanisms, but that understanding the variability of the angiogenic phenotypes may lead to design of more targeted anti-angiogenic therapies.

### 2.11. Host Immunity

Cancer immunotherapy is based on the fact that the host immune system detects abnormal cells and activates the cytotoxic potential of immune cells, especially tumour specific cytotoxic T cells, to eliminate cancer cells [225]. T cells target tumour cells by activation via dual signalling pathways. The first signal is through the T cell receptor (TCR) which recognises MHC-antigen on tumour cells. The second signal is induced by interaction of co-stimulatory factors, B7 molecule on the surface of antigen presenting cells (APC) and CD28 on the surface of T cells. Without either of these two signals, T cells cannot be activated [226]. Immune check points regulate the activation of T cells and initiation and termination of host immune responses.

Cytotoxic T lymphocyte antigen 4 (CTLA-4) is mainly expressed on T cells and to a lesser extent in active B cells, monocytes, granulocytes and dendritic cells (DCs). CTLA-4 is also expressed on regulatory T cells (Tregs) and produces the immunosuppressive molecule transforming growth factor-β (TGF-β) when activated with CD28 [227]. CTLA-4 (CD152) binds to B7 protein to induce T cell dysfunction and participate in negative regulation of the immune response. Under normal circumstances, the immunosuppressive effect of CTLA-4 is to stimulate the immune response effectively without excessive damage to normal tissues. However, cancer cells secrete TGF-β, which can stimulate the expression of CTLA-4, leading to T cell exhaustion [228]. T cell exhaustion is a state in which T cells have weak functions and might exert immunosuppression. The affinity of CD28 for CTLA-4 on the surface of T cells exceeds its affinity for the co-stimulatory molecules CD80 and CD86. Thus, T cells are prevented from proliferating and fail to function.

Programmed cell death protein-1 (PD-1) belongs to the CD28 receptor family and is mainly expressed on activated T cells and B cells. It is also found on monocytes and a small fraction of thymocytes. Ligands for PD-1 include programmed death-ligand 1 and 2 (PD-L1, PD-L2). Both are expressed on antigen-presenting cells, endothelial cells and activated lymphocytes [229]. PD-L1 is inducible and constitutively expressed in many malignancies despite limited expression in normal tissue, and its overexpression in malignant cells can promote tumour formation [230]. In melanoma and non-small-cell lung carcinoma (NSCLC), high expression of PD-L1 on tumour cells has been strongly associated with both high tumour grade and poor prognosis [231,232]. HNSCC tissues produce PD-L1 through an abnormal PD-1 signalling pathway, which leads to tumour immunosuppression [233], with the PD-1/PD-L1 pathway being activated in a chronic inflammatory environment. This axis contributes to the formation of HPV-positive HNSCC, whereby HPV-positive HNSCC tissues express more lymphocytes and higher levels of PD-L1 compared to HPV-negative HNSCC, while infiltrating CTLs express more PD-1 [234]. PD-L1 also delivers immunosuppressive signals by binding to T cells via the CD80 receptor. Depending on the HNSCC tumour immune environment, the PD-1/PD-L1 axis can be blocked by either targeting PD-1, thereby inhibiting its binding to PD-L1/PD-L2, or targeting PD-L1 to inhibit its binding to PD-1/CD80.

The PD-L1 signalling pathway not only downregulates anti-tumour T cell function but also affects cellular interactions between the innate and adaptive immune responses. Interactions between PD-1 and PD-L1 can regulate the tumour microenvironment by modulating the effects of T cells, DCs, myeloid-derived suppressor cells (MDSCs) and Tregs. Blocking PD-1 on Tregs thus inhibits their ability to mediate immune tolerance [235]. PD-1 expression on effector T cells increases sensitivity to the PD-L1 death signalling pathway.

## 3. Conclusions and Future Directions

Head and neck cancer is a highly heterogeneous tumour type, hence individual management should be based on both patient and tumour characteristics. To date, the optimal treatment for HNSCC patients involves a multidisciplinary approach including coordination of surgery, chemotherapy, radiation therapy and systemic therapies. Exploration of the molecular landscape of head and neck cancers utilising next generation sequencing has confirmed previously known HNSCC genome alterations such as mutations in *TP53*, *CDKN2A*, *PIK3CA*, *PTEN*, and *HRAS*, but also identified novel genes such as *NOTCH1*, *XRCC1*, and *RAD51C*. While *TP53* mutations are the most common genetic alterations in HNSCC, inactivation of p16 continues to be an important target in HPV-positive tumours, while exploration of DNA damage repair genes and synthetic lethality is emerging as a promising approach in combating HPV-negative tumours. Our ever-increasing understanding of etiological factors, tumour and patient characteristics, and the underlying molecular mutations in stratified tumours from different head and neck anatomical locations will continue to expand the development of therapeutic options against druggable targets to improve patient outcomes and control the personal and economic burden of head and neck cancer.

## References

[1] Kujan O., Huang G., Ravindran A., Vijayan M., Farah C.S. (2019). The role of cyclin-dependent kinases in oral potentially malignant disorders and oral squamous cell carcinoma. J. Oral Pathol. Med..

[2] Hashibe M., Boffetta P., Zaridze D., Shangina O., Szeszenia-Dabrowska N., Mates D., Fabianova E., Rudnai P., Brennan P. (2007). Contribution of tobacco and alcohol to the high rates of squamous cell carcinoma of the supraglottis and glottis in central europe. Am. J. Epidemiol..

[3] Hashibe M., Brennan P., Benhamou S., Castellsague X., Chu C., Curado M.P., Dal Maso L., Dauct A.W., Fabianova E., Wunsch V. (2007). Alcohol drinking in never users of tobacco, cigarette smoking in never drinkers, and the risk of head and neck cancer: Pooled analysis in the international head and neck cancer epidemiology consortium. J. Natl. Cancer Inst..

[4] Carrero I., Liu H.C., Sikora A.G., Milosavljevic A. (2019). Histoepigenetic analysis of HPV- and tobacco-associated head and neck cancer identifies both subtype-specific and common therapeutic targets despite divergent microenvironments. Oncogene.

[5] Liang X.H., Lewis J., Foote R., Smitb D., Kademani D. (2008). Prevalence and significance of human papillomavirus in oral tongue cancer: The Mayo Clinic experience. J. Oral Maxillofac. Surg..

[6] Chung C.H., Parker J.S., Karaca G., Wu J.Y., Funkhouser W.K., Moore’ D., Butterfoss D., Xiang D., Zonation A., Yin X.Y. (2004). Molecular classification of head and neck squamous cell carcinomas using patterns of gene expression. Cancer Cell.

[7] Dahlstrom K.R., Little J.A., Zafereo M.E., Lung M., Wei Q., Sturgis E.M. (2008). Squamous cell carcinoma of the head and neck in never smoker-never drinkers: A descriptive epidemiologic study. Head Neck J. Sci. Spec. Head Neck.

[8] Chung C.H., Gillison M.L. (2009). Human Papillomavirus in Head and Neck Cancer: Its Role in Pathogenesis and Clinical Implications. Clin. Cancer Res..

[9] Ziogas D.E., Katsios C.S., Tzaphlidou M., Roukos D.H. (2012). Targeted therapy: Overcoming drug resistance with clinical cancer genome. Expert Rev. Anticancer Ther..

[10] Ross J.S., Torres-Mora J., Wagle N., Jennings T.A., Jones D.M. (2010). Biomarker-based prediction of response to therapy for colorectal cancer: Current perspective. Am. J. Clin. Pathol..

[11] Mills G.B. (2012). An emerging toolkit for targeted cancer therapies. Genome Res..

[12] Simon R., Roychowdhury S. (2013). Implementing personalized cancer genomics in clinical trials. Nat. Rev. Drug Discov..

[13] Jessri M., Farah C.S. (2014). Harnessing Massively Parallel Sequencing in Personalized Head and Neck Oncology. J. Dent. Res..

[14] Fang W.J., Radovich M., Zhou A.W., Zheng Y.L., Zhao P., Huang W.Y., Mao C.Y., Zheng Y., Jia Y.K., Zheng S.S. (2014). ‘Druggable’ alterations detected by Ion Torrent in metastatic colorectal cancer patients. Oncol. Lett..

[15] Agrawal N., Frederick M.J., Pickering C.R., Bettegowda C., Chang K., Li R.J., Fakhry C., Xie T.X., Zhang J.X., Wang J. (2011). Exome Sequencing of Head and Neck Squamous Cell Carcinoma Reveals Inactivating Mutations in NOTCH1. Science.

[16] Stransky N., Egloff A.M., Tward A.D., Kostic A.D., Cibulskis K., Sivachenko A., Kryukov G.V., Lawrence M.S., Sougnez C., McKenna A. (2011). The Mutational Landscape of Head and Neck Squamous Cell Carcinoma. Science.

[17] Ciardiello F., Tortora G. (2008). Drug therapy: EGFR antagonists in cancer treatment. N. Engl. J. Med..

[18] Davies H., Bignell G.R., Cox C., Stephens P., Edkins S., Clegg S., Teague J., Woffendin H., Garnett M.J., Bottomley W. (2002). Mutations of the BRAF gene in human cancer. Nature.

[19] Paez J.G., Janne P.A., Lee J.C., Tracy S., Greulich H., Gabriel S., Herman P., Kaye F.J., Lindeman N., Boggon T.J. (2004). EGFR mutations in lung cancer: Correlation with clinical response to gefitinib therapy. Science.

[20] Samuels Y., Wang Z.H., Bardelli A., Silliman N., Ptak J., Szabo S., Yan H., Gazdar A., Powell D.M., Riggins G.J. (2004). High frequency of mutations of the PIK3CA gene in human cancers. Science.

[21] Stephens P., Hunter C., Bignell G., Edkins S., Davies H., Teague J., Stevens C., O’Meara S., Smith R., Parker A. (2004). Intragenic ERBB2 kinase mutations in tumours. Nature.

[22] Levine R.L., Wadleigh M., Cools J., Ebert B.L., Wernig G., Huntly B.J.P., Boggon T.J., Wlodarska L., Clark J.J., Moore S. (2005). Activating mutation in the tyrosine kinase JAK2 in polycythemia vera, essential thrombocythemia, and myeloid metaplasia with myelofibrosis. Cancer Cell.

[23] Jones S., Wang T.L., Shih I.M., Mao T.L., Nakayama K., Roden R., Glas R., Slamon D., Diaz L.A., Vogelstein B. (2010). Frequent Mutations of Chromatin Remodeling Gene ARID1A in Ovarian Clear Cell Carcinoma. Science.

[24] Leemans C.R., Braakhuis B.J.M., Brakenhoff R.H. (2011). The molecular biology of head and neck cancer. Nat. Rev. Cancer.

[25] Berenson J.R., Yang J., Mickel R.A. (1989). Frequent Amplification of the Bcl-1 Locus in Head and Neck Squamous-Cell Carcinomas. Oncogene.

[26] Loyo M., Li R.J., Bettegowda C., Pickering C.R., Frederick M.J., Myers J.N., Agrawal N. (2013). Lessons learned from next-generation sequencing in head and neck cancer. Head Neck J. Sci. Spec. Head Neck.

[27] Dotto G.P. (2008). Notch tumor suppressor function. Oncogene.

[28] Mahjabeen I., Masood N., Baig R.M., Sabir M., Inayat U., Malik F.A., Kayani M.A. (2012). Novel mutations of OGG1 base excision repair pathway gene in laryngeal cancer patients. Fam. Cancer.

[29] Scheckenbach K., Baldus S.E., Balz V., Freund M., Pakropa P., Sproll C., Schafer K.L., Wagenmann M., Schipper J., Hanenberg H. (2014). RAD51C—A new human cancer susceptibility gene for sporadic squamous cell carcinoma of the head and neck (HNSCC). Oral Oncol..

[30] Laborde R.R., Wang V.W., Smith T.M., Olson N.E., Olsen S.M., Garcia J.J., Olsen K.D., Moore E.J., Kasperbauer J.L., Tombers N.M. (2012). Transcriptional Profiling by Sequencing of Oropharyngeal Cancer. Mayo Clin. Proc..

[31] Poomsawat S., Sanguansin S., Punyasingh J., Vejchapipat P., Punyarit P. (2016). Expression of cdk6 in head and neck squamous cell carcinoma. Clin. Oral Investig..

[32] Neganova I., Lako M. (2008). G1 to S phase cell cycle transition in somatic and embryonic stem cells. J. Anat..

[33] Angadi P.V., Krishnapillai R. (2007). Cyclin D1 expression in oral squamous cell carcinoma and verrucous carcinoma: Correlation with histological differentiation. Oral Surg. Oral Med. Oral Pathol. Oral Radiol. Endod..

[34] Kordbacheh F., Farah C.S. (2021). Current and emerging molecular therapies for head and neck squamous cell carcinoma. Cancers.

[35] Suh Y., Amelio I., Guerrero Urbano T., Tavassoli M. (2014). Clinical update on cancer: Molecular oncology of head and neck cancer. Cell Death Dis..

[36] Pennell N.A., Lynch T.J. (2009). Combined inhibition of the VEGFR and EGFR signaling pathways in the treatment of NSCLC. Oncologist.

[37] Hynes N.E., Lane H.A. (2005). ERBB receptors and cancer: The complexity of targeted inhibitors. Nat. Rev. Cancer.

[38] Arienti C., Pignatta S., Tesei A. (2019). Epidermal Growth Factor Receptor Family and its Role in Gastric Cancer. Front. Oncol..

[39] Dienstmann R., De Dosso S., Felip E., Tabernero J. (2012). Drug development to overcome resistance to EGFR inhibitors in lung and colorectal cancer. Mol. Oncol..

[40] Lin S.Y., Makino K., Xia W., Matin A., Wen Y., Kwong K.Y., Bourguignon L., Hung M.C. (2001). Nuclear localization of EGF receptor and its potential new role as a transcription factor. Nat. Cell Biol..

[41] Rao V.H., Kandel A., Lynch D., Pena Z., Marwaha N., Deng C., Watson P., Hansen L.A. (2012). A positive feedback loop between HER2 and ADAM12 in human head and neck cancer cells increases migration and invasion. Oncogene.

[42] Cavalot A., Martone T., Roggero N., Brondino G., Pagano M., Cortesina G. (2007). Prognostic impact of HER-2/neu expression on squamous head and neck carcinomas. Head Neck J. Sci. Spec. Head Neck.

[43] Ekberg T., Nestor M., Engstrom M., Nordgren H., Wester K., Carlsson J., Anniko M. (2005). Expression of EGFR, HER2, HER3, and HER4 in metastatic squamous cell carcinomas of the oral cavity and base of tongue. Int. J. Oncol..

[44] Kriegs M., Clauditz T.S., Hoffer K., Bartels J., Buhs S., Gerull H., Zech H.B., Bussmann L., Struve N., Rieckmann T. (2019). Analyzing expression and phosphorylation of the EGF receptor in HNSCC. Sci. Rep..

[45] Grandis J.R., Tweardy D.J. (1993). Elevated Levels of Transforming Growth-Factor-Alpha and Epidermal Growth-Factor Receptor Messenger-Rna Are Early Markers of Carcinogenesis in Head and Neck-Cancer. Cancer Res..

[46] Loeffler-Ragy J., Witsch-Baumgartner M., Tzankov A., Hilbe W., Schwentner I., Sprinzl G.M., Utermann G., Zwierzina H. (2006). Low incidence of mutations in EGFR kinase domain in Caucasian patients with head and neck squamous cell carcinoma. Eur. J. Cancer.

[47] Temam S., Kawaguchi H., El-Naggar A.K., Jelinek J., Tang H.L., Liu D.D., Lang W.H., Issa J.P., Lee J.J., Mao L. (2007). Epidermal growth factor receptor copy number alterations correlate with poor clinical outcome in patients with head and neck squamous cancer. J. Clin. Oncol..

[48] Sok J.C., Coppelli F.M., Thomas S.M., Lango M.N., Xi S.C., Hunt J.L., Freilino M.L., Graner M.W., Wikstrand C.J., Bigner D.D. (2006). Mutant epidermal growth factor receptor (EGFRvIII) contributes to head and neck cancer growth and resistance to EGFR targeting. Clin. Cancer Res..

[49] Licitra L., Mesia R., Rivera F., Remenar E., Hitt R., Erfan J., Rottey S., Kawecki A., Zabolotnyy D., Benasso M. (2011). Evaluation of EGFR gene copy number as a predictive biomarker for the efficacy of cetuximab in combination with chemotherapy in the first-line treatment of recurrent and/or metastatic squamous cell carcinoma of the head and neck: EXTREME study. Ann. Oncol..

[50] Chung C.H., Ely K., McGavran L., Varella-Garcia M., Parker J., Parker N., Jarrett C., Carter J., Murphy B.A., Netterville J. (2006). Increased epidermal growth factor receptor gene copy number is associated with poor prognosis in head and neck squamous cell carcinomas. J. Clin. Oncol..

[51] Moon C., Chae Y.K., Lee J. (2010). Targeting epidermal growth factor receptor in head and neck cancer: Lessons learned from cetuximab. Exp. Biol. Med..

[52] Engelman J.A. (2009). Targeting PI3K signalling in cancer: Opportunities, challenges and limitations. Nat. Rev. Cancer.

[53] Liu P.X., Cheng H.L., Roberts T.M., Zhao J.J. (2009). Targeting the phosphoinositide 3-kinase pathway in cancer. Nat. Rev. Drug Discov..

[54] Fruman D.A., Chiu H., Hopkins B.D., Bagrodia S., Cantley L.C., Abraham R.T. (2017). The PI3K Pathway in Human Disease. Cell.

[55] Owonikoko T.K., Khuri F.R. (2013). Targeting the PI3K/AKT/mTOR pathway: Biomarkers of success and tribulation. Am. Soc. Clin. Oncol. Educ. Book.

[56] Freudlsperger C., Burnett J.R., Friedman J.A., Kannabiran V.R., Chen Z., Van Waes C. (2011). EGFR-PI3K-AKT-mTOR signaling in head and neck squamous cell carcinomas: Attractive targets for molecular-oriented therapy. Expert Opin. Ther. Targets.

[57] Psyrri A., Seiwert T.Y., Jimeno A. (2013). Molecular pathways in head and neck cancer: EGFR, PI3K, and more. Am. Soc. Clin. Oncol. Educ Book.

[58] Gougis P., Moreau Bachelard C., Kamal M., Gan H.K., Borcoman E., Torossian N., Bieche I., Le Tourneau C. (2019). Clinical Development of Molecular Targeted Therapy in Head and Neck Squamous Cell Carcinoma. JNCI Cancer Spectr..

[59] Martin C.L., Reshmi S.C., Ried T., Gottberg W., Wilson J.W., Reddy J.K., Khanna P., Johnson J.T., Myers E.N., Gollin S.M. (2008). Chromosomal imbalances in oral squamous cell carcinoma: Examination of 31 cell lines and review of the literature. Oral Oncol..

[60] Fenic I., Steger K., Gruber C., Arens C., Woenckhaus J. (2007). Analysis of PIK3CA and Akt/protein kinase B in head and neck squamous cell carcinoma. Oncol. Rep..

[61] Woenckhaus J., Steger K., Werner E., Fenic I., Gamerdinger U., Dreyer T., Stahl U. (2002). Genomic gain of PIK3CA and increased expression of p110alpha are associated with progression of dysplasia into invasive squamous cell carcinoma. J. Pathol..

[62] Estilo C.L., Pornchai O., Ngai I., Patel S.G., Reddy P.G., Dao S., Shaha A.R., Kraus D.H., Boyle J.O., Wong R.J. (2003). The role of novel oncogenes squamous cell carcinoma-related oncogene and phosphatidylinositol 3-kinase p110alpha in squamous cell carcinoma of the oral tongue. Clin. Cancer Res..

[63] Cancer Genome Atlas Network (2015). Comprehensive genomic characterization of head and neck squamous cell carcinomas. Nature.

[64] Morris L.G., Taylor B.S., Bivona T.G., Gong Y., Eng S., Brennan C.W., Kaufman A., Kastenhuber E.R., Banuchi V.E., Singh B. (2011). Genomic dissection of the epidermal growth factor receptor (EGFR)/PI3K pathway reveals frequent deletion of the EGFR phosphatase PTPRS in head and neck cancers. Proc. Natl. Acad. Sci. USA.

[65] Weber A., Langhanki L., Sommerer F., Markwarth A., Wittekind C., Tannapfel A. (2003). Mutations of the BRAF gene in squamous cell carcinoma of the head and neck. Oncogene.

[66] Yoon S., Seger R. (2006). The extracellular signal-regulated kinase: Multiple substrates regulate diverse cellular functions. Growth Factors.

[67] Rong C., Muller M.F., Xiang F., Jensen A., Weichert W., Major G., Plinkert P.K., Hess J., Affolter A. (2020). Adaptive ERK signalling activation in response to therapy and in silico prognostic evaluation of EGFR-MAPK in HNSCC. Br. J. Cancer.

[68] Takacs T., Kudlik G., Kurilla A., Szeder B., Buday L., Vas V. (2020). The effects of mutant Ras proteins on the cell signalome. Cancer Metastasis Rev..

[69] Bos J.L. (1989). Ras Oncogenes in Human Cancer—A Review. Cancer Res..

[70] Wellbrock C., Karasarides M., Marais R. (2004). The RAF proteins take centre stage. Nat. Rev. Mol. Cell Biol..

[71] Peyssonnaux C., Eychene A. (2001). The Raf/MEK/ERK pathway: New concepts of activation. Biol. Cell.

[72] Guo Y.J., Pan W.W., Liu S.B., Shen Z.F., Xu Y., Hu L.L. (2020). ERK/MAPK signalling pathway and tumorigenesis. Exp. Ther. Med..

[73] Matthaios D., Zarogoulidis P., Balgouranidou I., Chatzaki E., Kakolyris S. (2011). Molecular Pathogenesis of Pancreatic Cancer and Clinical Perspectives. Oncology.

[74] Santarpia L., Lippman S.M., El-Naggar A.K. (2012). Targeting the MAPK-RAS-RAF signaling pathway in cancer therapy. Expert Opin. Ther. Targets.

[75] Poulikakos P.I., Rosen N. (2011). Mutant BRAF melanomas—Dependence and resistance. Cancer Cell.

[76] Xing M. (2007). BRAF mutation in papillary thyroid cancer: Pathogenic role, molecular bases, and clinical implications. Endocr. Rev..

[77] Garnett M.J., Rana S., Paterson H., Barford D., Marais R. (2005). Wild-type and mutant B-RAF activate C-RAF through distinct mechanisms involving heterodimerization. Mol. Cell.

[78] Andersson E.R., Sandberg R., Lendahl U. (2011). Notch signaling: Simplicity in design, versatility in function. Development.

[79] Tchekneva E.E., Goruganthu M.U.L., Uzhachenko R.V., Thomas P.L., Antonucci A., Chekneva I., Koenig M., Piao L., Akhter A., de Aquino M.T.P. (2019). Correction to: Determinant roles of dendritic cell-expressed Notch Delta-like and Jagged ligands on anti-tumor T-cell immunity. J. Immunother. Cancer.

[80] Bray S.J. (2006). Notch signalling: A simple pathway becomes complex. Nat. Rev. Mol. Cell Biol..

[81] Kovall R.A. (2008). More complicated than it looks: Assembly of Notch pathway transcription complexes. Oncogene.

[82] Kopan R., Ilagan M.X.G. (2009). The Canonical Notch Signaling Pathway: Unfolding the Activation Mechanism. Cell.

[83] Steinbuck M.P., Winandy S. (2018). A Review of Notch Processing With New Insights Into Ligand-Independent Notch Signaling in T-Cells. Front. Immunol..

[84] Ellisen L.W., Bird J., West D.C., Soreng A.L., Reynolds T.C., Smith S.D., Sklar J. (1991). Tan-1, the Human Homolog of the Drosophila Notch Gene, Is Broken by Chromosomal Translocations in T-Lymphoblastic Neoplasms. Cell.

[85] Weng A.P., Ferrando A.A., Lee W., Morris J.P., Silverman L.B., Sanchez-Irizarry C., Blacklow S.C., Look A.T., Aster J.C. (2004). Activating mutations of NOTCH1 in human T cell acute lymphoblastic leukemia. Science.

[86] Puente X.S., Pinyol M., Quesada V., Conde L., Ordonez G.R., Villamor N., Escaramis G., Jares P., Bea S., Gonzalez-Diaz M. (2011). Whole-genome sequencing identifies recurrent mutations in chronic lymphocytic leukaemia. Nature.

[87] Pinto I., Duque M., Goncalves J., Akkapeddi P., Oliveira M.L., Cabrita R., Yunes J.A., Durum S.K., Barata J.T., Fragoso R. (2020). NRARP displays either pro- or anti-tumoral roles in T-cell acute lymphoblastic leukemia depending on Notch and Wnt signaling. Oncogene.

[88] Liu Y.F., Chiang S.L., Lin C.Y., Chang J.G., Chung C.M., Ko A.M., Lin Y.Z., Lee C.H., Lee K.W., Chen M.K. (2016). Somatic Mutations and Genetic Variants of NOTCH1 in Head and Neck Squamous Cell Carcinoma Occurrence and Development. Sci. Rep..

[89] Pickering C.R., Zhang J.X., Yoo S.Y., Bengtsson L., Moorthy S., Neskey D.M., Zhao M., Alves M.V.O., Chang K., Drummond J. (2013). Integrative Genomic Characterization of Oral Squamous Cell Carcinoma Identifies Frequent Somatic Drivers. Cancer Discov..

[90] Kolev V., Mandinova A., Guinea-Viniegra J., Hu B., Lefort K., Lambertini C., Neel V., Dummer R., Wagner E.F., Dotto G.P. (2008). EGFR signalling as a negative regulator of Notch1 gene transcription and function in proliferating keratinocytes and cancer. Nat. Cell Biol..

[91] Dotto G.P. (2009). Crosstalk of Notch with p53 and p63 in cancer growth control. Nat. Rev. Cancer.

[92] Talora C., Sgroi D.C., Crum C.P., Dotto G.P. (2002). Specific down-modulation of Notch1 signaling in cervical cancer cells is required for sustained HPV-E6/E7 expression and late steps of malignant transformation. Genes Dev..

[93] Organ S.L., Tsao M.S. (2011). An overview of the c-MET signaling pathway. Ther. Adv. Med. Oncol..

[94] Kim E.S., Salgia R. (2009). MET Pathway as a Therapeutic Target. J. Thorac. Oncol..

[95] Ma P.C., Maulik G., Christensen J., Salgia R. (2003). c-Met: Structure, functions and potential for therapeutic inhibition. Cancer Metastasis Rev..

[96] Peruzzi B., Bottaro D.P. (2006). Targeting the c-Met signaling pathway in cancer. Clin. Cancer Res..

[97] Hammond D.E., Urbe S., Woude G.F.V., Clague M.J. (2001). Down-regulation of MET, the receptor for hepatocyte growth factor. Oncogene.

[98] Zhang Y., Xia M., Jin K., Wang S., Wei H., Fan C., Wu Y., Li X., Li X., Li G. (2018). Function of the c-Met receptor tyrosine kinase in carcinogenesis and associated therapeutic opportunities. Mol. Cancer.

[99] Ma P.C., Jagadeeswaran R., Jagadeesh S., Tretiakova M.S., Nallasura V., Fox E.A., Hansen M., Schaefer E., Naoki K., Lader A. (2005). Functional expression and mutations of c-met and its therapeutic inhibition with SU11274 and small interfering RNA in non-small cell lung cancer. Cancer Res..

[100] Ariyawutyakorn W., Saichaemchan S., Varella-Garcia M. (2016). Understanding and Targeting MET Signaling in Solid Tumors—Are We There Yet?. J. Cancer.

[101] Knowles L.M., Stabile L.P., Egloff A.M., Rothstein M.E., Thomas S.M., Gubish C.T., Lerner E.C., Seethala R.R., Suzuki S., Quesnelle K.M. (2009). HGF and c-Met Participate in Paracrine Tumorigenic Pathways in Head and Neck Squamous Cell Cancer. Clin. Cancer Res..

[102] Seiwert T.Y., Jagadeeswaran R., Faoro L., Janamanchi V., Nallasura V., El Dinali M., Yala S., Kanteti R., Cohen E.E.W., Lingen M.W. (2009). The MET Receptor Tyrosine Kinase Is a Potential Novel Therapeutic Target for Head and Neck Squamous Cell Carcinoma. Cancer Res..

[103] Vsiansky V., Gumulec J., Raudenska M., Masarik M. (2018). Prognostic role of c-Met in head and neck squamous cell cancer tissues: A meta-analysis. Sci. Rep..

[104] Marur S., D’Souza G., Westra W.H., Forastiere A.A. (2010). HPV-associated head and neck cancer: A virus-related cancer epidemic. Lancet Oncol..

[105] Zhao D., Wang S.H., Feng Y., Hua C.G., Zhao J., Tang X.F. (2011). Intratumoral c-Met expression is associated with vascular endothelial growth factor C expression, lymphangiogenesis, and lymph node metastasis in oral squamous cell carcinoma: Implications for use as a prognostic marker. Hum. Pathol..

[106] Lo Muzio L., Farina A., Rubini C., Coccia E., Capogreco M., Colella G., Leonardi R., Campisi G., Carinci F. (2006). Effect of c-Met expression on survival in head and neck squamous cell carcinoma. Tumor Biol..

[107] Di Renzo M.F., Olivero M., Martone T., Maffe A., Maggiora P., Stefani A.D., Valente G., Giordano S., Cortesina G., Comoglio P.M. (2000). Somatic mutations of the MET oncogene are selected during metastatic spread of human HNSC carcinomas. Oncogene.

[108] Jagadeeswaran R., Ma P.C., Seiwert T.Y., Jagadeeswaran S., Zumba O., Nallasura V., Ahmed S., Filiberti R., Paganuzzi M., Puntoni R. (2006). Functional analysis of c-Met/hepatocyte growth factor pathway in malignant pleural mesothelioma. Cancer Res..

[109] Aaronson D.S., Horvath C.M. (2002). A road map for those who don’t know JAK-STAT. Science.

[110] Kisseleva T., Bhattacharya S., Braunstein J., Schindler C.W. (2002). Signaling through the JAK/STAT pathway, recent advances and future challenges. Gene.

[111] Lai S.Y., Johnson F.M. (2010). Defining the role of the JAK-STAT pathway in head and neck and thoracic malignancies: Implications for future therapeutic approaches. Drug Resist. Updates.

[112] Villarino A.V., Kanno Y., O’Shea J.J. (2017). Mechanisms and consequences of Jak-STAT signaling in the immune system. Nat. Immunol..

[113] Siddiquee K.A.Z., Turkson J. (2008). STAT3 as a target for inducing apoptosis in solid and hematological tumors. Cell Res..

[114] Rawlings J.S., Rosler K.M., Harrison D.A. (2004). The JAK/STAT signaling pathway. J. Cell Sci..

[115] Foster F.M., Traer C.J., Abraham S.M., Fry M.J. (2003). The phosphoinositide (PI) 3-kinase family. J. Cell Sci..

[116] Wu J., Patmore D.M., Jousma E., Eaves D.W., Breving K., Patel A.V., Schwartz E.B., Fuchs J.R., Cripe T.P., Stemmer-Rachamimov A.O. (2014). EGFR-STAT3 signaling promotes formation of malignant peripheral nerve sheath tumors. Oncogene.

[117] Quesnelle K.M., Boehm A.L., Grandis J.R. (2007). STAT-mediated EGFR signaling in cancer. J. Cell. Biochem..

[118] Andersen P., Pedersen M.W., Woetmann A., Villingshoj M., Stockhausen M.T., Odum N., Poulsen H.S. (2008). EGFR induces expression of IRF-1 via STAT1 and STAT3 activation leading to growth arrest of human cancer cells. Int. J. Cancer.

[119] Lierman E., Michaux L., Beullens E., Pierre P., Marynen P., Cools J., Vandenberghe P. (2009). FIP1L1-PDGFR alpha D842V, a novel panresistant mutant, emerging after treatment of FIP1L1-PDGFR alpha T674I eosinophilic leukemia with single agent sorafenib. Leukemia.

[120] Olayioye M.A., Beuvink I., Horsch K., Daly J.M., Hynes N.E. (1999). ErbB receptor-induced activation of Stat transcription factors is mediated by Src tyrosine kinases. J. Biol. Chem..

[121] Parsons S.J., Parsons J.T. (2004). Src family kinases, key regulators of signal transduction. Oncogene.

[122] Sansone P., Bromberg J. (2012). Targeting the Interleukin-6/Jak/Stat Pathway in Human Malignancies. J. Clin. Oncol..

[123] Carbajo-Pescador S., Ordonez R., Benet M., Jover R., Garcia-Palomo A., Mauriz J.L., Gonzalez-Gallego J. (2013). Inhibition of VEGF expression through blockade of Hif1 alpha and STAT3 signalling mediates the anti-angiogenic effect of melatonin in HepG2 liver cancer cells. Br. J. Cancer.

[124] Jung J.E., Lee H.G., Cho I.H., Chung D.H., Yoon S.H., Yang Y.M., Lee J.W., Choi S., Park J.W., Ye S.K. (2005). STAT3 is a potential modulator of HIF-1-mediated VEGF expression in human renal carcinoma cells. FASEB J..

[125] Itoh M., Murata T., Suzuki T., Shindoh M., Nakajima K., Imai K., Yoshida K. (2006). Requirement of STAT3 activation for maximal collagenase-1 (MMP-1) induction by epidermal growth factor and malignant characteristics in T24 bladder cancer cells. Oncogene.

[126] Lee J.W., Kwak H.J., Lee J.J., Kim Y.N., Lee J.W., Park M.J., Jung S.E., Hong S.I., Lee J.H., Lee J.S. (2008). HSP27 regulates cell adhesion and invasion via modulation of focal adhesion kinase and MMP-2 expression. Eur. J. Cell Biol..

[127] Ghosh M.C., Grass L., Soosaipillai A., Sotiropoulou G., Diamandis E.P. (2004). Human kallikrein 6 degrades extracellular matrix proteins and may enhance the metastatic potential of tumour cells. Tumor Biol..

[128] Quintero-Fabian S., Arreola R., Becerril-Villanueva E., Torres-Romero J.C., Arana-Argaez V., Lara-Riegos J., Ramirez-Camacho M.A., Alvarez-Sanchez M.E. (2019). Role of Matrix Metalloproteinases in Angiogenesis and Cancer. Front. Oncol..

[129] Niu G.L., Wright K.L., Ma Y.H., Wright G.M., Huang M., Irby R., Briggs J., Karras J., Cress W.D., Pardoll D. (2005). Role of Stat3 in regulating p53 expression and function. Mol. Cell. Biol..

[130] Yu H., Kortylewski M., Pardoll D. (2007). Crosstalk between cancer and immune cells: Role of STAT3 in the tumour microenvironment. Nat. Rev. Immunol..

[131] Ivashkiv L.B., Donlin L.T. (2014). Regulation of type I interferon responses. Nat. Rev. Immunol..

[132] Larsen L., Ropke C. (2002). Suppressors of cytokine signalling: SOCS. Apmis.

[133] He B., You L., Uematsu K., Zang K.L., Xu Z.D., Lee A.Y., Costello J.F., McCormick F., Jablons D.M. (2003). SOCS-3 is frequently silenced by hypermethylation and suppresses cell growth in human lung cancer. Proc. Natl. Acad. Sci. USA.

[134] Weber A., Hengge U.R., Bardenheuer W., Tischoff I., Sommerer F., Markwarth A., Dietz A., Wittekind C., Tannapfel A. (2005). SOCS-3 is frequently methylated in head and neck squamous cell carcinoma and its precursor lesions and causes growth inhibition. Oncogene.

[135] Lee T.L., Yeh J., Van Waes C., Chen Z. (2006). Epigenetic modification of SOCS-1 differentially regulates STAT3 activation in response to interleukin-6 receptor and epidermal growth factor receptor signaling through JAK and/or MEK in head and neck squamous cell carcinomas. Mol. Cancer Ther..

[136] Grandis J.R., Drenning S.D., Zeng Q., Watkins S.C., Melhem M.F., Endo S., Johnson D.E., Huang L., He Y., Kim J.D. (2000). Constitutive activation of Stat3 signaling abrogates apoptosis in squamous cell carcinogenesis in vivo. Proc. Natl. Acad. Sci. USA.

[137] Kijima T., Niwa H., Steinman R.A., Drenning S.D., Gooding W.E., Wentzel A.L., Xi S., Grandis J.R. (2002). STAT3 activation abrogates growth factor dependence and contributes to head and neck squamous cell carcinoma tumor growth in vivo. Cell Growth Differ..

[138] Zhang Q., Thomas S.M., Lui V.W., Xi S., Siegfried J.M., Fan H., Smithgall T.E., Mills G.B., Grandis J.R. (2006). Phosphorylation of TNF-alpha converting enzyme by gastrin-releasing peptide induces amphiregulin release and EGF receptor activation. Proc. Natl. Acad. Sci. USA.

[139] Zhang Q., Thomas S.M., Xi S., Smithgall T.E., Siegfried J.M., Kamens J., Gooding W.E., Grandis J.R. (2004). SRC family kinases mediate epidermal growth factor receptor ligand cleavage, proliferation, and invasion of head and neck cancer cells. Cancer Res..

[140] Sen B., Saigal B., Parikh N., Gallick G., Johnson F.M. (2009). Sustained Src inhibition results in signal transducer and activator of transcription 3 (STAT3) activation and cancer cell survival via altered Janus-activated kinase-STAT3 binding. Cancer Res..

[141] Koppikar P., Lui V.W., Man D., Xi S., Chai R.L., Nelson E., Tobey A.B., Grandis J.R. (2008). Constitutive activation of signal transducer and activator of transcription 5 contributes to tumor growth, epithelial-mesenchymal transition, and resistance to epidermal growth factor receptor targeting. Clin. Cancer Res..

[142] Stancato L.F., David M., Carter-Su C., Larner A.C., Pratt W.B. (1996). Preassociation of STAT1 with STAT2 and STAT3 in separate signalling complexes prior to cytokine stimulation. J. Biol Chem..

[143] Leong P.L., Xi S., Drenning S.D., Dyer K.F., Wentzel A.L., Lerner E.C., Smithgall T.E., Grandis J.R. (2002). Differential function of STAT5 isoforms in head and neck cancer growth control. Oncogene.

[144] Xi S., Zhang Q., Gooding W.E., Smithgall T.E., Grandis J.R. (2003). Constitutive activation of Stat5b contributes to carcinogenesis in vivo. Cancer Res..

[145] Lai S.Y., Childs E.E., Xi S., Coppelli F.M., Gooding W.E., Wells A., Ferris R.L., Grandis J.R. (2005). Erythropoietin-mediated activation of JAK-STAT signaling contributes to cellular invasion in head and neck squamous cell carcinoma. Oncogene.

[146] Sen B., Peng S., Woods D.M., Wistuba I., Bell D., El-Naggar A.K., Lai S.Y., Johnson F.M. (2012). STAT5A-mediated SOCS2 expression regulates Jak2 and STAT3 activity following c-Src inhibition in head and neck squamous carcinoma. Clin. Cancer Res..

[147] Sherr C.J., McCormick F. (2002). The RB and p53 pathways in cancer. Cancer Cell.

[148] Bensaad K., Vousden K.H. (2007). p53: New roles in metabolism. Trends Cell Biol..

[149] Hussain S.P., Harris C.C. (2006). p53 biological network: At the crossroads of the cellular-stress response pathway and molecular carcinogenesis. J. Nippon Med. Sch..

[150] Honda R., Tanaka H., Yasuda H. (1997). Oncoprotein MDM2 is a ubiquitin ligase E3 for tumor suppressor p53. FEBS Lett..

[151] Vogelstein B., Lane D., Levine A.J. (2000). Surfing the p53 network. Nature.

[152] O’Driscoll M., Ruiz-Perez V.L., Woods C.G., Jeggo P.A., Goodship J.A. (2003). A splicing mutation affecting expression of ataxia-telangiectasia and Rad3-related protein (ATR) results in Seckel syndrome. Nat. Genet..

[153] Mei L., Zhang J., He K., Zhang J. (2019). Ataxia telangiectasia and Rad3-related inhibitors and cancer therapy: Where we stand. J. Hematol. Oncol..

[154] Nakano K., Vousden K.H. (2001). PUMA, a novel proapoptotic gene, is induced by p53. Mol. Cell.

[155] Schuler M., Maurer U., Goldstein J.C., Breitenbucher F., Hoffarth S., Waterhouse N.J., Green D.R. (2003). p53 triggers apoptosis in oncogene-expressing fibroblasts by the induction of Noxa and mitochondrial Bax translocation. Cell Death Differ..

[156] Gartel A.L., Tyner A.L. (2002). The role of the cyclin-dependent kinase inhibitor p21 in apoptosis. Mol. Cancer Ther..

[157] Kreis N.N., Louwen F., Yuan J. (2019). The Multifaceted p21 (Cip1/Waf1/CDKN1A) in Cell Differentiation, Migration and Cancer Therapy. Cancers.

[158] Allocati N., Di Ilio C., De Laurenzi V. (2012). p63/p73 in the control of cell cycle and cell death. Exp. Cell Res..

[159] Cino E.A., Soares I.N., Pedrote M.M., de Oliveira G.A., Silva J.L. (2016). Aggregation tendencies in the p53 family are modulated by backbone hydrogen bonds. Sci. Rep..

[160] Das S.K., Hashimoto T., Shimizu K., Yoshida T., Sakai T., Sowa Y., Komoto A., Kanazawa K. (2005). Fucoxanthin induces cell cycle arrest at G(0)/G(1) phase in human colon carcinoma cells through up-regulation of p21(WAF1/Cip1). Biochim. Biophys. Acta-Gen. Subj..

[161] Moser J., Miller I., Carter D., Spencer S.L. (2018). Control of the Restriction Point by Rb and p21. Proc. Natl. Acad. Sci. USA.

[162] Poeta M.L., Manola J., Goldwasser M.A., Forastiere A., Benoit N., Califano J.A., Ridge J.A., Goodwin J., Kenady D., Saunders J. (2007). TP53 mutations and survival in squamous-cell carcinoma of the head and neck. N. Engl. J. Med..

[163] Friend S. (1994). P53—A Glimpse at the Puppet Behind the Shadow Play. Science.

[164] Brennan J.A., Mao L., Hruban R.H., Boyle J.O., Eby Y.J., Koch W.M., Goodman S.N., Sidransky D. (1995). Molecular Assessment of Histopathological Staging in Squamous-Cell Carcinoma of the Head and Neck. N. Engl. J. Med..

[165] Ebrahimi M., Boldrup L., Coates P.J., Wahlin Y.B., Bourdon J.C., Nylander K. (2008). Expression of novel p53 isoforms in oral lichen planus. Oral Oncol..

[166] Forastiere A., Koch W., Trotti A., Sidransky D. (2001). Medical progress—Head and neck cancer. N. Engl. J. Med..

[167] Boyle J.O., Hakim J., Koch W., Vanderriet P., Hruban R.H., Roa R.A., Correo R., Eby Y.J., Ruppert J.M., Sidransky D. (1993). The Incidence of P53 Mutations Increases with Progression of Head and Neck-Cancer. Cancer Res..

[168] Klinakis A., Rampias T. (2020). TP53 mutational landscape of metastatic head and neck cancer reveals patterns of mutation selection. EBioMedicine.

[169] Skinner H.D., Sandulache V.C., Ow T.J., Meyn R.E., Yordy J.S., Beadle B.M., Fitzgerald A.L., Giri U., Ang K.K., Myers J.N. (2012). TP53 Disruptive Mutations Lead to Head and Neck Cancer Treatment Failure through Inhibition of Radiation-Induced Senescence. Clin. Cancer Res..

[170] Morton J.P., Timpson P., Karim S.A., Ridgway R.A., Athineos D., Doyle B., Jamieson N.B., Oien K.A., Lowy A.M., Brunton V.G. (2010). Mutant p53 drives metastasis and overcomes growth arrest/senescence in pancreatic cancer. Proc. Natl. Acad. Sci. USA.

[171] Kandoth C., McLellan M.D., Vandin F., Ye K., Niu B.F., Lu C., Xie M.C., Zhang Q.Y., McMichael J.F., Wyczalkowski M.A. (2013). Mutational landscape and significance across 12 major cancer types. Nature.

[172] Caponio V.C.A., Troiano G., Adipietro I., Zhurakivska K., Arena C., Mangieri D., Mascitti M., Cirillo N., Lo Muzio L. (2020). Computational analysis of TP53 mutational landscape unveils key prognostic signatures and distinct pathobiological pathways in head and neck squamous cell cancer. Br. J. Cancer.

[173] Bova R.J., Quinn D.I., Nankervis J.S., Cole I.E., Sheridan B.F., Jensen M.J., Morgan G.J., Hughes C.J., Sutherland R.L. (1999). Cyclin D1 and p16(INK4A) expression predict reduced survival in carcinoma of the anterior tongue. Clin. Cancer Res..

[174] Smeets S.J., Braakhuis B.J.M., Abbas S., Snijders P.J.F., Ylstra B., van de Wiel M.A., Meijer G.A., Leemans C.R., Brakenhoff R.H. (2006). Genome-wide DNA copy number alterations in head and neck squamous cell carcinomas with or without oncogene-expressing human papillomavirus. Oncogene.

[175] Mao L., Lee J.S., Fan Y.H., Ro J.Y., Batsakis J.G., Lippman S., Hittelman W., Hong W.K. (1996). Frequent microsatellite alterations at chromosomes 9p21 and 3p14 in oral premalignant lesions and their value in cancer risk assessment. Nat. Med..

[176] Lee D.J., Schonleben F., Banuchi V.E., Qiu W.L., Close L.G., Assaad A.M., Su G.H. (2010). Multiple tumor-suppressor genes on chromosome 3p contribute to head and neck squamous cell carcinoma tumorigenesis. Cancer Biol. Ther..

[177] Rosin M.P., Cheng X., Poh C., Lam W.L., Huang Y.Q., Lovas J., Berean K., Epstein J.B., Priddy R., Le N.D. (2000). Use of allelic loss to predict malignant risk for low-grade oral epithelial dysplasia. Clin. Cancer Res..

[178] Lee J.J., Hong W.K., Hittelman W.N., Mao L., Lotan R., Shin D.M., Benner S.E., Xu X.C., Lee J.S., Papadimitrakopoulou V.M. (2000). Predicting cancer development in oral leukoplakia: Ten years of translational research. Clin. Cancer Res..

[179] Perez-Sayans M., Suarez-Penaranda J.M., Gayoso-Diz P., Barros-Angueira F., Gandara-Rey J.M., Garcia-Garcia A. (2011). p16(INK4a)/CDKN2 expression and its relationship with oral squamous cell carcinoma is our current knowledge enough?. Cancer Lett..

[180] Smeets S.J., Brakenhoff R.H., Ylstra B., van Wieringen W.N., van de Wiel M.A., Leemans C.R., Braakhuis B.J.M. (2009). Genetic classification of oral and oropharyngeal carcinomas identifies subgroups with a different prognosis. Cell. Oncol..

[181] Thomas M., Narayan N., Pim D., Tomaic V., Massimi P., Nagasaka K., Kranjec C., Gammoh N., Banks L. (2008). Human papillomaviruses, cervical cancer and cell polarity. Oncogene.

[182] Lingen M.W., Xiao W.H., Schmitt A., Jiang B., Pickard R., Kreinbrink P., Perez-Ordonez B., Jordan R.C., Gillison M.L. (2013). Low etiologic fraction for high-risk human papillomavirus in oral cavity squamous cell carcinomas. Oral Oncol..

[183] Conway M.J., Meyers C. (2009). Replication and Assembly of Human Papillomaviruses. J. Dent. Res..

[184] zur Hausen H. (2002). Papillomaviruses and cancer: From basic studies to clinical application. Nat. Rev. Cancer.

[185] Kreimer A.R., Clifford G.M., Boyle P., Franceschi S. (2005). Human papillomavirus types in head and neck squamous cell carcinomas worldwide: A systematic review. Cancer Epidemiol. Biomark. Prev..

[186] Scheffner M., Werness B.A., Huibregtse J.M., Levine A.J., Howley P.M. (1990). The E6 Oncoprotein Encoded by Human Papillomavirus Type-16 and Type-18 Promotes the Degradation of P53. Cell.

[187] Thomas M., Pim D., Banks L. (1999). The role of the E6-p53 interaction in the molecular pathogenesis of HPV. Oncogene.

[188] Wiest T., Schwarz E., Enders C., Flechtenmacher C., Bosch F.X. (2002). Involvement of intact HPV16 E6/E7 gene expression in head and neck cancers with unaltered p53 status and perturbed pRb cell cycle control. Oncogene.

[189] Benevolo M., Mottolese M., Marandino F., Vocaturo G., Sindico R., Piperno G., Mariani L., Sperduti I., Canalini P., Donnorso R.P. (2006). Immunohistochemical expression of p16(INK4a) is predictive of HR-HPV infection in cervical low-grade lesions. Mod. Pathol..

[190] Lechner M., Frampton G.M., Fenton T., Feber A., Palmer G., Jay A., Pillay N., Forster M., Cronin M.T., Lipson D. (2013). Targeted next-generation sequencing of head and neck squamous cell carcinoma identifies novel genetic alterations in HPV+ and HPV- tumors. Genome Med..

[191] Stephen J.K., Divine G., Chen K.M., Chitale D., Havard S., Worsham M.J. (2013). Significance of p16 in Site-specific HPV Positive and HPV Negative Head and Neck Squamous Cell Carcinoma. Cancer Clin. Oncol..

[192] Poling J.S., Ma X.J., Bui S., Luo Y., Li R., Koch W.M., Westra W.H. (2014). Human papillomavirus (HPV) status of non-tobacco related squamous cell carcinomas of the lateral tongue. Oral Oncol..

[193] Nurse P. (1990). Universal control mechanism regulating onset of M-phase. Nature.

[194] Hanken H., Grobe A., Cachovan G., Smeets R., Simon R., Sauter G., Heiland M., Blessmann M. (2014). CCND1 amplification and cyclin D1 immunohistochemical expression in head and neck squamous cell carcinomas. Clin. Oral Investig..

[195] Feng L., Xie Y., Zhang H., Wu Y. (2012). miR-107 targets cyclin-dependent kinase 6 expression, induces cell cycle G1 arrest and inhibits invasion in gastric cancer cells. Med. Oncol..

[196] Todorova T.A., Jordanov S.H., Stancheva G.S., Chalakov I.J., Melnicharov M.B., Kunev K.V., Mitev V.I., Kaneva R.P., Goranova T.E. (2015). Mutational Status of CDKN2A and TP53 Genes in Laryngeal Squamous Cell Carcinoma. Pathol. Oncol. Res..

[197] Ben-Dayan M.M., Ow T.J., Belbin T.J., Wetzler J., Smith R.V., Childs G., Diergaarde B., Hayes D.N., Grandis J.R., Prystowsky M.B. (2017). Nonpromoter methylation of the CDKN2A gene with active transcription is associated with improved locoregional control in laryngeal squamous cell carcinoma. Cancer Med..

[198] Jiang X., Ye J., Dong Z., Hu S., Xiao M. (2019). Novel genetic alterations and their impact on target therapy response in head and neck squamous cell carcinoma. Cancer Manag. Res..

[199] Bartkova J., Horejsi Z., Sehested M., Nesland J.M., Rajpert-De Meyts E., Skakkebaek N.E., Stucki M., Jackson S., Lukas J., Bartek J. (2007). DNA damage response mediators MDC1 and 53BP1: Constitutive activation and aberrant loss in breast and lung cancer, but not in testicular germ cell tumours. Oncogene.

[200] Weber A.M., Ryan A.J. (2015). ATM and ATR as therapeutic targets in cancer. Pharm. Ther..

[201] Xia B., Sheng Q., Nakanishi K., Ohashi A., Wu J., Christ N., Liu X., Jasin M., Couch F.J., Livingston D.M. (2006). Control of BRCA2 cellular and clinical functions by a nuclear partner, PALB2. Mol. Cell.

[202] Chung C.H., Guthrie V.B., Masica D.L., Tokheim C., Kang H., Richmon J., Agrawal N., Fakhry C., Quon H., Subramaniam R.M. (2015). Genomic alterations in head and neck squamous cell carcinoma determined by cancer gene-targeted sequencing. Ann. Oncol..

[203] Seiwert T.Y., Zuo Z., Keck M.K., Khattri A., Pedamallu C.S., Stricker T., Brown C., Pugh T.J., Stojanov P., Cho J. (2015). Integrative and comparative genomic analysis of HPV-positive and HPV-negative head and neck squamous cell carcinomas. Clin. Cancer Res..

[204] Vaupel P., Kallinowski F., Okunieff P. (1989). Blood-Flow, Oxygen and Nutrient Supply, and Metabolic Microenvironment of Human-Tumors—A Review. Cancer Res..

[205] Jordan B.F., Sonveaux P. (2012). Targeting tumor perfusion and oxygenation to improve the outcome of anticancer therapy. Front. Pharm..

[206] Nordsmark M., Bentzen S.M., Rudat V., Brizel D., Lartigau E., Stadler P., Becker A., Adam M., Molls M., Dunst J. (2005). Prognostic value of tumor oxygenation in 397 head and neck tumors after primary radiation therapy. An international multi-center study. Radiother. Oncol..

[207] Alsahafi E., Begg K., Amelio I., Raulf N., Lucarelli P., Sauter T., Tavassoli M. (2019). Clinical update on head and neck cancer: Molecular biology and ongoing challenges. Cell Death Dis..

[208] Kaelin W.G. (2005). Proline hydroxylation and gene expression. Annu. Rev. Biochem..

[209] Park S.K., Dadak A.M., Haase V.H., Fontana L., Giaccia A.J., Johnson R.S. (2003). Hypoxia-induced gene expression occurs solely through the action of hypoxia-inducible factor 1 alpha (HIF-1 alpha): Role of cytoplasmic trapping of HIF-2 alpha. Mol. Cell. Biol..

[210] Kyzas P.A., Stefanou D., Batistatou A., Agnantis N.J. (2005). Hypoxia-induced tumor angiogenic pathway in head and neck cancer: An in vivo study. Cancer Lett..

[211] Wang X., Schneider A. (2010). HIF-2 alpha-mediated activation of the epidermal growth factor receptor potentiates head and neck cancer cell migration in response to hypoxia. Carcinogenesis.

[212] Thomas G.V., Tran C., Mellinghoff I.K., Welsbie D.S., Chan E., Fueger B., Czernin J., Sawyers C.L. (2006). Hypoxia-inducible factor determines sensitivity to inhibitors of mTOR in kidney cancer. Nat. Med..

[213] Christopoulos A., Ahn S.M., Klein J.D., Kim S. (2011). Biology of Vascular Endothelial Growth Factor and Its Receptors in Head and Neck Cancer: Beyond Angiogenesis. Head Neck J. Sci. Spec. Head Neck.

[214] Hoeben A., Landuyt B., Highley M.S., Wildiers H., Van Oosterom A.T., De Bruijn E.A. (2004). Vascular endothelial growth factor and angiogenesis. Pharmacol. Rev..

[215] Carla C., Daris F., Cecilia B., Francesca B., Francesca C., Paolo F. (2012). Angiogenesis in head and neck cancer: A review of the literature. J. Oncol..

[216] Claffey K.P., Shih S.C., Mullen A., Dziennis S., Cusick J.L., Abrams K.R., Lee S.W., Detmar M. (1998). Identification of a human VPF/VEGF 3’ untranslated region mediating hypoxia-induced mRNA stability. Mol. Biol Cell.

[217] Pugh C.W., Ratcliffe P.J. (2003). Regulation of angiogenesis by hypoxia: Role of the HIF system. Nat. Med..

[218] Harris A.L. (2002). Hypoxia—A key regulatory factor in tumour growth. Nat. Rev. Cancer.

[219] Kim C.Y., Tsai M.H., Osmanian C., Graeber T.G., Lee J.E., Giffard R.G., DiPaolo J.A., Peehl D.M., Giaccia A.J. (1997). Selection of human cervical epithelial cells that possess reduced apoptotic potential to low-oxygen conditions. Cancer Res..

[220] Lopez-Ocejo O., Viloria-Petit A., Bequet-Romero M., Mukhopadhyay D., Rak J., Kerbel R.S. (2000). Oncogenes and tumor angiogenesis: The HPV-16 E6 oncoprotein activates the vascular endothelial growth factor (VEGF) gene promoter in a p53 independent manner. Oncogene.

[221] Soengas M.S., Alarcon R.M., Yoshida H., Giaccia A.J., Hakem R., Mak T.W., Lowe S.W. (1999). Apaf-1 and caspase-9 in p53-dependent apoptosis and tumor inhibition. Science.

[222] Marsden V.S., O’Connor L., O’Reilly L.A., Silke J., Metcalf D., Ekert P.G., Huang D.C., Cecconi F., Kuida K., Tomaselli K.J. (2002). Apoptosis initiated by Bcl-2-regulated caspase activation independently of the cytochrome c/Apaf-1/caspase-9 apoptosome. Nature.

[223] Suzuki H., Tomida A., Tsuruo T. (2001). Dephosphorylated hypoxia-inducible factor 1 alpha as a mediator of p53-dependent apoptosis during hypoxia. Oncogene.

[224] Hasina R., Whipple M.E., Martin L.E., Kuo W.P., Ohno-Machado L., Lingen M.W. (2008). Angiogenic heterogeneity in head and neck squamous cell carcinoma: Biological and therapeutic implications. Lab. Investig..

[225] Kalbasi A., Ribas A. (2020). Tumour-intrinsic resistance to immune checkpoint blockade. Nat. Rev. Immunol..

[226] Mei Z., Huang J., Qiao B., Lam A.K. (2020). Immune checkpoint pathways in immunotherapy for head and neck squamous cell carcinoma. Int. J. Oral Sci..

[227] Walker L.S. (2013). Treg and CTLA-4: Two intertwining pathways to immune tolerance. J. Autoimmun..

[228] Ravi R., Noonan K.A., Pham V., Bedi R., Zhavoronkov A., Ozerov I.V., Makarev E., Artemov A.V., Wysocki P.T., Mehra R. (2018). Bifunctional immune checkpoint-targeted antibody-ligand traps that simultaneously disable TGFbeta enhance the efficacy of cancer immunotherapy. Nat. Commun..

[229] de Vicente J.C., Rodriguez-Santamarta T., Rodrigo J.P., Blanco-Lorenzo V., Allonca E., Garcia-Pedrero J.M. (2019). PD-L1 Expression in Tumor Cells Is an Independent Unfavorable Prognostic Factor in Oral Squamous Cell Carcinoma. Cancer Epidemiol. Biomark. Prev..

[230] Yu W., Hua Y., Qiu H., Hao J., Zou K., Li Z., Hu S., Guo P., Chen M., Sui S. (2020). PD-L1 promotes tumor growth and progression by activating WIP and beta-catenin signaling pathways and predicts poor prognosis in lung cancer. Cell Death Dis..

[231] Madore J., Strbenac D., Vilain R., Menzies A.M., Yang J.Y., Thompson J.F., Long G.V., Mann G.J., Scolyer R.A., Wilmott J.S. (2016). PD-L1 Negative Status is Associated with Lower Mutation Burden, Differential Expression of Immune-Related Genes, and Worse Survival in Stage III Melanoma. Clin. Cancer Res..

[232] Mu C.Y., Huang J.A., Chen Y., Chen C., Zhang X.G. (2011). High expression of PD-L1 in lung cancer may contribute to poor prognosis and tumor cells immune escape through suppressing tumor infiltrating dendritic cells maturation. Med. Oncol..

[233] Zandberg D.P., Strome S.E. (2014). The role of the PD-L1:PD-1 pathway in squamous cell carcinoma of the head and neck. Oral Oncol..

[234] Lyford-Pike S., Peng S., Young G.D., Taube J.M., Westra W.H., Akpeng B., Bruno T.C., Richmon J.D., Wang H., Bishop J.A. (2013). Evidence for a role of the PD-1:PD-L1 pathway in immune resistance of HPV-associated head and neck squamous cell carcinoma. Cancer Res..

[235] Wang W., Lau R., Yu D., Zhu W., Korman A., Weber J. (2009). PD1 blockade reverses the suppression of melanoma antigen-specific CTL by CD4+ CD25(Hi) regulatory T cells. Int. Immunol..

